# Association of Waist Circumference Gain and Incident Prediabetes Defined by Fasting Glucose: A Seven-Year Longitudinal Study in Beijing, China

**DOI:** 10.3390/ijerph14101208

**Published:** 2017-10-10

**Authors:** Li-Xin Tao, Kun Yang, Fang-Fang Huang, Xiang-Tong Liu, Xia Li, Yan-Xia Luo, Li-Juan Wu, Xiu-Hua Guo

**Affiliations:** 1 School of Public Health, Capital Medical University, Beijing 100069, China; taolixin@ccmu.edu.cn (L.-X.T.); yangkun_1123@163.com (K.Y.); hff87hff87@163.com (F.-F.H.); lxiangtong@163.com (X.-T.L.); lyx100@ccmu.edu.cn (Y.-X.L.); wujuan811017@163.com (L.-J.W.); 2 Beijing Municipal Key Laboratory of Clinical Epidemiology, Capital Medical University, Beijing 100069, China; 3 Department of Mathematics and Statistics, La Trobe University, Melbourne 3086, Australia; lixia_new@163.com

**Keywords:** waist circumference gain, prediabetes, abdominal obesity, type 2 diabetes

## Abstract

The risk of incident prediabetes with gain in waist circumference (WC) has not been addressed among Chinese adults. A total of 7951 participants who underwent health check-ups at the Beijing Physical Examination Center and Beijing Xiaotangshan hospital were recruited in 2009 and followed up in 2016. Participants were classified into four groups according to categories of percent WC gain: ≤−2.5%, −2.5–2.5%, 2.5–5%, and >5%. The effect of WC gain on prediabetes was evaluated using modified Poisson regression models. Over seven years of follow-up, we identified 1034 prediabetes cases (413 women). Compared with a WC gain of ≤−2.5%, participants with a WC gain of >5% have a higher risk of prediabetes, be they male (non-abdominal obesity at baseline group: RR = 1.57, 95% CI: 1.10–2.24, abdominal obesity at baseline group: RR = 1.66, 95% CI: 1.20–2.30) or female (non-abdominal obesity at baseline group: RR = 1.74, 95% CI: 1.14–2.64, abdominal obesity at baseline group: RR = 2.47, 95% CI: 1.43–4.28). In conclusion, the risk of prediabetes increased significantly with increasing WC for both genders in the Chinese population. Lifestyle interventions aiming at preventing abdominal obesity are urgently needed to reduce the increasing burden of prediabetes, diabetes, and its complications.

## 1. Introduction

China has the world’s largest diabetes epidemic and the prevalence of diabetes and prediabetes continue to increase [1]. The prevalence of diabetes in China was reported to be 0.67% in 1980 and significantly increased to 10.90% according to the most recent study in 2013 [2,3]. Moreover, in the 2013 survey, the prevalence of prediabetes was estimated to be 35.70%, implying that approximately 356 million Chinese adults are living with prediabetes [3]. Prediabetes is also a pressing clinical and public health problem in China. Prediabetes is a condition with which a person has a higher risk of developing diabetes and its associated complications, and 5–10% of those with prediabetes can progress to diabetes every year [4]. Almost 70% of individuals with prediabetes will eventually develop diabetes according to the American Diabetes Association (ADA) expert panel. In the China Da Qing Diabetes Prevention Study, the 23-year cumulative incidence of diabetes was 89.9% among controls with an impaired glucose tolerance (IGT) defined with repeated oral glucose tolerance tests (OGTTs) even after a six-year lifestyle intervention [5]. A large number of trials have demonstrated obvious reductions in the risk of diabetes development among individuals with prediabetes after lifestyle or pharmacological interventions [6,7,8,9,10,11,12]. Prediabetes can convert back to normoglycemia and diabetes can be prevented by changes in lifestyles. In the Ely study of the natural history of diabetes in England found that 55–80% of the participants with impaired fasting glucose (IFG) at baseline were normoglycemic after 10-year follow-up [13]. Observational evidence has shown the associations between prediabetes and increased risk of chronic kidney disease, diabetic peripheral neuropathy, diabetic retinopathy, and some macrovascular disease [14,15,16,17]. Further studies are, therefore, needed to identify modifiable lifestyle contributors to prediabetes.

Obesity has been a major global health challenge and it has become an epidemic in China in recent decades [18,19]. The prevalence of abdominal obesity is increasing in the Chinese population. Previous evidence confirmed that abdominal obesity was significantly and positively associated with all-cause cardiovascular disease (CVD) and cancer mortality [20,21] independently of general obesity. Moreover, abdominal obesity has been recommended as a better predictor for the risk of myocardial infarction [22] and type 2 diabetes [23]. Waist circumference (WC) and body mass index (BMI) are two anthropometric measures for obesity commonly used in routine health check-ups. However, there was evidence implying that abdominal obesity measured by WC may be a better indicator of obesity in terms of risk of diabetes than overall obesity measured by BMI or another discriminator [24,25]. However, most previous studies accounted for the effect of WC based on a cutoff point on incident diabetes [25,26]. Hence, whether WC dynamic change plays an important role in development of diabetes or prediabetes has not been well documented. Only two studies in Japan have assessed the relationship between WC change and diabetes risk [27,28]. The studies showed that monitoring WC gain is effective in preventing type 2 diabetes occurrence in the Japanese population, especially for individuals with a high WC. However, the effect of long-term WC gain on prediabetes incidence has not been established.

Therefore, we aimed to assess the risk of prediabetes with seven-year changes in abdominal obesity as measured by WC in Chinese adults and whether the relationship differs by gender.

## 2. Subjects and Methods

### 2.1. Subjects

This longitudinal cohort consisted of 16,717 participants attended health check-ups in Beijing Physical Examination Center and Beijing Xiaotangshan hospital in 2009 at baseline and 2016 at follow-up. Among the 16,717 participants, we excluded those with a previous diagnosis of CVD (*n* = 228), cerebrovascular disease (CBVD) (*n* = 102), or cancer (*n* = 95), those with diabetes or prediabetes at baseline (*n* = 5037), those with diabetes or with drug treatment for diabetes at follow-up (*n* = 157), and those with incomplete data to calculate WC D-value (*n* = 3147). Finally, we had data for 3906 men and 4045 women. Therefore, all participants were free of diabetes, prediabetes, CVD, CBVD, and cancer at baseline in 2009. The study was approved by the Ethics Committee of Capital Medical University (no: 2013SY26), and all procedures were performed in accordance with the 1964 Declaration of Helsinki and its later amendments or with comparable ethical standards.

Questionnaire interviews and anthropometric and laboratory measurements were performed at baseline and follow-up with the consent of all participants. The difference value (D-value) in WC was calculated by subtracting the WC measured at baseline in 2009 from that measured at follow-up in 2016.

### 2.2. Measurement of Biochemical and Clinical Variables

The participants underwent physical examinations that included the measurement of height, weight, WC, blood pressure (BP), and overnight fasting blood sampling. Height was measured, to the nearest 0.5 cm, without shoes, with the participant’s back square against the wall tape, eyes looking straight ahead with a right-angle triangle resting on the scalp and against the wall. Weight was measured with a lever balance to the nearest 100 g and without shoes. WC was measured with gentle breathing at the midpoint between the lowest rib and the iliac crest. The cutoff point of WC for abdominal obesity is 90 cm for men and 80 cm for women according to the International Diabetes Federation Epidemiology Task Force Consensus Group [29]. BP was measured for each subject in sitting position after a 5 min rest period. During the 30 min preceding the measurements, the subjects were required to refrain from smoking or caffeine. Three readings each of systolic and diastolic BPs were recorded, with an interval of at least 1 min, and the average of the last two measurements was used for data analysis. Several cuffs of different sizes are available to obtain accurate BP measurements in the hospital. A standard mercury sphygmomanometer was used with one of four cuff sizes (pediatric, regular adult, large adult, or thigh) based on the participant’s arm circumference in the hospital.

Blood samples were collected from subjects after an overnight fast of at least 12 h. Total cholesterol (TC), high density lipoprotein cholesterol (HDLC), triglycerides (TG), and fasting plasma glucose (FPG) were measured by an enzymatic method using a chemistry analyzer (Beckman LX 20, Beckman, Brea, CA, USA) at the central laboratory of the two hospitals. All analyses were performed in accordance with the manufacturer’s recommendations.

Information of lifestyle factors, including education level, physical activity, smoking status, and drinking status were collected using a self-administered questionnaire. Smoking was defined as currently smoking and/or having smoked at least 100 cigarettes in one’s lifetime. Drinking was defined as having consumed alcohol 12 or more times in the last year. Physical activity was classified as less than once every week, more than once every week, and more than once every day.

The same measurements as for the baseline examination were taken in the seven-year follow-up visit. Prediabetes was defined as any participants who did not have diabetes but who had an FPG level in a range from 5.6 mmol/L (100 mg/dL) to 6.9 mmol/L (125 mg/dL) according to the ADA 2010 criteria [30].

### 2.3. Statistical Analyses

Data were presented as mean ± SD for normal distributed continuous variables and median (interquartile range) for skewed distributed continuous variables. Categorical variables were described as number (%). The characteristics of the participants were compared among groups. Percent WC gain >0% indicated that WC increased from baseline to follow-up: the greater the percent WC gain, the greater the increase in WC. The percent WC gain was classified as follows: ≤−2.5%, –2.5–2.5%, 2.5–5%, and >5% [31]. We investigated the prediabetes risk according to the above WC levels. One-way analysis of variance was used for normal distributive continuous variables and Kruskal–Wallis rank tests were used for skewed distributive continuous variables among four groups. Using a WC gain of ≤−2.5% as the reference group, Tukey’s tests were used to compare the levels of the continuous variables between the other three groups and the reference group. Chi-square tests were used for nominal categorical variables, and Kruskal–Wallis rank tests were used for ordinal categorical variables among four groups.

Subjects were stratified by gender and abdominal or non-abdominal obesity at baseline. In all primary analyses, a WC gain of ≤−2.5% was considered as the reference group. Modified Poisson regression analyses were performed to estimate the adjusted relative risks (RRs) and 95% confidence intervals (CIs) of WC gain for risk of prediabetes [32]. All potential confounding variables in the current regression analyses were collected at baseline. Model 1 was initially age-adjusted. Then, Model 2 was adjusted for age, education level, smoking, drinking status, physical activity, and family history of type 2 diabetes at baseline. Lastly, based on Model 2, Model 3 was additionally adjusted for systolic blood pressure (SBP), diastolic blood pressure (DBP), and levels of FPG, TC, TG, and HDLC at baseline.

All statistical analyses were performed using SAS software (Version 9.2; SAS Institute, Chicago, IL, USA), and *p* < 0.05 was considered statistically significant.

## 3. Results

### 3.1. Characteristics of Study Participants

A total of 7951 participants were eligible for inclusion in this study. The median ages were 39 years old for men and 37 years old for women at baseline. The baseline characteristics of the study population by WC gain category are in Table 1. Age, WC, SBP, DBP, TC, and TG were significantly associated with WC gain (all *p* < 0.05).

### 3.2. Association between WC Gain and Risk of Prediabetes

During a seven-year follow-up, 1034 prediabetes cases (413 women) were identified. To examine the association between WC dynamic change and prediabetes risk in detail, the range of percent WC gain was divided into four categories. In all models, the RRs for prediabetes increased significantly with increasing percent WC gain for both genders (all *p* for trend <0.05).

We stratified participants by abdominal obesity or not at baseline (Table 2). The prevalence of abdominal obesity increased from 26.47 to 34.46% for men and from 12.76 to 21.83% for women from baseline to follow-up. Significantly positive linear trends in risk of prediabetes were observed with increasing WC gain for men and women regardless of their abdominal obesity status at baseline (all *p* for trend <0.05). Male participants with a WC gain of >5%, compared with those with a WC gain of ≤−2.5%, have a higher risk of prediabetes (non-abdominal obesity at baseline: RR = 1.57, 95% CI: 1.10–2.24, abdominal obesity at baseline: RR = 1.66, 95% CI: 1.20–2.30). Female participants with a WC gain of >5%, compared with those with a WC gain of ≤−2.5%, have a higher risk of prediabetes (non-abdominal obesity at baseline: RR = 1.74, 95% CI: 1.14–2.64, abdominal obesity at baseline: RR = 2.47, 95% CI: 1.43–4.28).

We also examined the association of absolute values of WC gain and prediabetes risk by genders (see Table S1). In all three models, the RRs for prediabetes increased significantly with increasing percent WC gain for both genders (all *p* for trend <0.05).

### 3.3. Association between WC Gain and Prediabetes by Transition in Abdominal Obesity Status

Moreover, we assessed the risk of prediabetes by the transition in abdominal obesity status (Table 3). Notably, as compared with participants who were not abdominally obese at both baseline and follow-up, the risk of prediabetes was significantly greater for both genders who were abdominally obese at follow-up regardless of their abdominal obesity status at baseline. In Model 3, male participants who were obese at follow-up, compared with those who were not abdominally obese at both baseline and follow-up, were at a higher risk of prediabetes (non-abdominal obesity at baseline: RR = 1.36, 95% CI: 1.09–1.69, abdominal obesity at baseline: RR = 1.35, 95% CI: 1.11–1.63). Female participants who were obese at follow-up, compared with those who were not abdominally obese at both baseline and follow-up, were also at a higher risk of prediabetes (non-abdominal obesity at baseline: RR = 1.60, 95% CI: 1.23–2.08, abdominal obesity at baseline: RR = 1.85, 95% CI: 1.42–2.41).

## 4. Discussion

To our knowledge, this is the first longitudinal study that investigate the long-term effect of WC dynamic gain on the incidence of prediabetes among Chinese adults. Abdominal obesity is prevalent in China and is significantly positively associated with a high risk of prediabetes. A dose–response relationship between changes of WC and incident prediabetes for both genders, regardless of abdominal obesity status at baseline after adjustment for lifestyle and personal factors, as well as levels of lipid markers at baseline, were investigated in the present study.

The prevalence of obesity is increasing at an alarming rate in China with the rapid development of China. The unhealthy dietary and lack of exercise and physical activity patterns lead to an increase in the prevalence of obesity. The China Health and Nutrition Surveys illustrated that the prevalence of abdominal obesity increased from 8.5 to 27.8% for men and from 27.8 to 45.9% for women during the period from 1993 to 2009 [19]. According to the CHPSNE study in 2009, the prevalence of abdominal obesity was 37.6% in a representative sample of urban adults in Northeast China [33]. Our study confirmed that abdominal obesity is seriously prevalent in urban China and positively associated with a high risk of prediabetes. Therefore, strategies aiming at preventing abdominal obesity are urgently needed to reduce the increasing burden of diabetes, CVD, and metabolic diseases.

Previous studies have assessed the relationship between abdominal obesity and diabetes incidence [24,25,26,27,28,29,30,33,34]. Nevertheless, whether the long-term dynamic increase in WC leads to prediabetes is not well described. Similar to prior longitudinal population-based studies about obesity and diabetes, we found a strong positive association between WC gain and prediabetes. The Suita Study in an urban area of Japan demonstrated that preventing WC gain is important in preventing type 2 diabetes in the Japanese population, especially among participants with a relatively high level of WC [28]. WC gain can reflect a specific accumulation of visceral adipose tissue, which may cause insulin resistance, contributing to type 2 diabetes. A previous study reported that effective WC reduction rate can reduce the risk of development of type 2 diabetes in non-diabetic Japanese men with abdominal obesity [28]. The Newcastle Group’s study indicated the remission of type 2 diabetes when abdominal fat is drastically reduced by the use of low-calorie diet. Weight loss by sustainable use of low-calorie diet brought about normalization of liver fat content and insulin sensitivity [35]. The SABE survey: Health, Wellness, and Aging study indicated that WC has been associated with type 2 diabetes, regardless of gender, age, ethnicity, and BMI [34]. A prospective cohort study (Health Professionals Follow-Up Study) of 27,270 men indicated that WC is a strong and independent predictor for the risk of type 2 diabetes [24]. However, these studies were mostly based on the relation between absolute value gains in WC and diabetes. The different WC level at baseline may have a different effect on the risk of type 2 diabetes with the same values gain. The cut-off points for WC are inevitably arbitrary. Only one study assessed the association between the percent WC gain and incident diabetes [28]. Moreover, no study has investigated the association between percent gain in WC and the risk of prediabetes.

Our study has several strengths. It is the first to evaluate the relationship between the dynamic change of WC and prediabetes in Chinese adults. The second strength is that the study has a longitudinal, prospective design, which can help to estimate the causal relationship between WC gain and prediabetes. Finally, all participants were free of prediabetes at baseline, which can affirm the true relationship between WC gain and prediabetes. There was also a limitation to our study: since information on lifestyles were self-reported, measurement errors are inevitable. The other limitation is that the adjustment of lifestyle factors as covariates were only limited to baseline factors and not changes over time. Future studies could examine changes in lifestyle factors with WC changes in predicting risk of pre-diabetes in the population.

## 5. Conclusions

The risk of prediabetes increased significantly with increasing WC for both genders in the Chinese population. Strategies aiming at preventing abdominal obesity are urgently needed to reduce the increasing burden of prediabetes, diabetes, and other complications.

## Figures and Tables

**Table 1:** Baseline characteristics of study participants stratified by percent waist circumference (WC) gain.

Gender	Baseline Characteristic	Percent WC Gain (%)				*p-*Value
		≤−2.5	−2.5 to 2.5	2.5 to 5	>5	
Men	*n*	697	1103	595	1511	
	Age (years)	45 (34–55)	43 (33–52) *	40 (30–52) *	34 (27–46) *	<0.0001
	WC (cm)	90 (83–97)	88 (83–92) *	85 (80–90) *	80 (75–87) *	<0.0001
	SBP (mmHg)	120 (110–130)	120 (110–130) *	120 (110–128) *	120 (110–124) *	<0.0001
	DBP (mmHg)	80 (72–84)	80 (70–84) *	80 (70–82) *	80 (70–80) *	<0.0001
	FPG (mmol/L)	5.22 (5.00–5.42)	5.22 (4.99–5.41)	5.21 (4.98–5.40)	5.20 (4.99–5.39)	0.1563
	TC (mmol/L)	4.98 (4.41–5.53)	4.90 (4.36–5.49) *	4.80 (4.25–5.38)	4.64 (4.11–5.24) *	<0.0001
	TG (mmol/L)	1.41 (0.96–2.07)	1.35 (0.91–2.06) *	1.23 (0.86–1.88)	1.08 (0.76–1.66) *	<0.0001
	HDLC (mmol/L)	1.19 (1.03–1.37)	1.18 (1.01–1.36) *	1.18 (1.04–1.36)	1.22 (1.06–1.40) *	0.0005
	College or higher education (*n*, %)	681 (97.70)	1076 (97.55)	577 (96.97)	1480 (97.95)	0.6108
	Physical activity (*n*, %)					0.0198
	less than once every week	99 (63.06)	137 (51.70)	77 (49.68)	176 (45.6)	
	more than once every week	580 (83.21)	922 (83.59)	491 (82.52)	1246 (82.46)	
	more than once every day	18 (11.46)	44 (16.6)	27 (17.42)	89 (23.06)	
	Smoking status (*n*, %)	42 (6.03)	84 (7.62)	55 (9.24)	121 (8.01)	0.1768
	Drinking status (*n*, %)	96 (13.77)	179 (16.23)	109 (18.32)	224 (14.82)	0.1052
	Family history of type 2 diabetes (*n*, %)	32 (4.59)	58 (5.26)	34 (5.71)	75 (4.96)	0.8127
Women	*n*	585	792	608	2060	
	Age (years)	40 (31–51)	41 (32–50) *	38 (31–46)	33 (28–43) *	<0.0001
	WC (cm)	76 (71–80)	73 (68–80) *	70 (66–76)	67 (63–73) *	<0.0001
	SBP (mmHg)	110 (100–120)	110 (100–120) *	106 (100–113)	106 (100–110) *	<0.0001
	DBP (mmHg)	70 (70–80)	70 (70–80) *	70 (66–76)	70 (66–74) *	<0.0001
	FPG (mmol/L)	5.06 (4.85–5.28)	5.12 (4.90–5.32) *	5.11 (4.90–5.32)	5.06 (4.85–5.28) *	0.0003
	TC (mmol/L)	4.79 (4.18–5.50)	4.79 (4.24–5.46) *	4.63 (4.12–5.23)	4.51 (4.00–5.16) *	<0.0001
	TG (mmol/L)	0.94 (0.64–1.38)	0.89 (0.60–1.30) *	0.84 (0.60–1.22)	0.76 (0.56–1.10) *	<0.0001
	HDLC (mmol/L)	1.45 (1.27–1.69)	1.45 (1.25–1.68)	1.45 (1.26–1.65)	1.47 (1.29–1.69)	0.1456
	College or higher education (*n*, %)	577 (98.63)	775 (97.85)	596 (98.03)	2013 (97.72)	0.5886
	Physical activity (*n*, %)					0.4131
	less than once every week	107 (74.83)	145 (77.13)	131 (71.20)	438 (71.34)	
	more than once every week	468 (80.00)	637 (80.43)	462 (75.99)	1577 (76.55)	
	more than once every day	10 (6.99)	10 (5.32)	15 (8.15)	45 (7.33)	
	Smoking status (*n*, %)	4 (0.68)	6 (0.76)	1 (0.16)	9 (0.44)	0.3878
	Drinking status (*n*, %)	26 (4.44)	29 (3.66)	31 (5.10)	81 (3.93)	0.5260
	Family history of type 2 diabetes (*n*, %)	39 (6.67)	40 (5.05)	42 (6.91)	127 (6.17)	0.4681
Total	*n*	1282	1895	1203	3571	
	Age (years)	43 (32–53)	42 (33–51) *	39 (30–49)	34 (28–44) *	<0.0001
	WC (cm)	83 (76–92)	83 (74–89) *	78 (69–85)	73 (66–80) *	<0.0001
	SBP (mmHg)	116 (108–126)	118 (110–126) *	110 (100–120)	110 (100–120) *	<0.0001
	DBP (mmHg)	76 (70–80)	76 (70–80) *	72 (70–80)	70 (70–80) *	<0.0001
	FPG (mmol/L)	5.15 (4.93–5.37)	5.18 (4.95–5.38) *	5.16 (4.93–5.36)	5.12 (4.9–5.33) *	<0.0001
	TC (mmol/L)	4.91 (4.31–5.51)	4.85 (4.3–5.48) *	4.7 (4.2–5.32)	4.57 (4.04–5.21) *	<0.0001
	TG (mmol/L)	1.16 (0.76–1.78)	1.16 (0.77–1.76) *	1.01 (0.68–1.56)	0.88 (0.62–1.33) *	<0.0001
	HDLC (mmol/L)	1.3 (1.10–1.53)	1.28 (1.09–1.5)	1.31 (1.13–1.54)	1.36 (1.17–1.59)	<0.0001
	College or higher education (*n*, %)	1258 (98.13)	1851 (97.68)	1173 (97.51)	3493 (97.82)	0.7425
	Physical activity (*n*, %)					0.5005
	less than once every week	206 (16.07)	282 (14.88)	208 (17.29)	614 (17.19)	
	more than once every week	1048 (81.75)	1559 (82.27)	953 (79.22)	2823 (79.05)	
	more than once every day	28 (2.18)	54 (2.85)	42 (3.49)	134 (3.75)	
	Smoking status (*n*, %)	46 (3.59)	90 (4.75)	56 (4.66)	130 (3.64)	0.1237
	Drinking status (*n*, %)	122 (9.52)	208 (10.98)	140 (11.64)	305 (8.54)	0.0027
	Family history of type 2 diabetes (*n*, %)	71 (5.54)	98 (5.17)	76 (6.32)	202 (5.66)	0.6048

WC: waist circumference; SBP: systolic blood pressure; DBP: diastolic blood pressure; FPG: fasting plasma glucose; TC: total cholesterol; TG: triglyceride; HDLC: high-density lipoprotein cholesterol. Data are median (interquartile range) or *n* (%). * *p* < 0.05 vs. percent WC gain ≤−2.5%.

**Table 2:** Risk of prediabetes by percent WC gain.

Gender	Percent WC Gain (%)	Total	Prediabetes (*n*, %)	RR (95% CI)		
				Model 1 *	Model 2 ^†^	Model 3 ^‡^
Men	Non-abdominal obesity at baseline					
	≤−2.5	347	39 (11.24)	1.00	1.00	1.00
	−2.5 to 2.5	665	81 (12.18)	1.16 (0.81–1.67)	1.17 (0.81–1.67)	1.14 (0.77–1.70)
	2.5 to 5	442	62 (14.03)	1.35 (0.93–1.98)	1.35 (0.93–1.97)	1.29 (0.85–1.94)
	>5	1250	178 (14.24)	1.52 (1.08–2.13)	1.54 (1.10–2.16)	1.57 (1.10–2.24)
	*p* for trend			0.0041	0.0025	0.0020
	Abdominal obesity at baseline					
	≤−2.5	350	64 (18.29)	1.00	1.00	1.00
	−2.5 to 2.5	438	89 (20.32)	1.11 (0.83–1.48)	1.11 (0.83–1.48)	1.14 (0.82–1.57)
	2.5 to 5	153	36 (23.53)	1.30 (0.90–1.86)	1.30 (0.90–1.86)	1.34 (0.89–2.02)
	>5	261	72 (27.59)	1.54 (1.15–2.07)	1.54 (1.15–2.07)	1.66 (1.20–2.30)
	*p* for trend			0.0032	0.0028	0.0016
Women	Non-abdominal obesity at baseline					
	≤−2.5	404	26 (6.44)	1.00	1.00	1.00
	−2.5 to 2.5	578	38 (6.57)	1.06 (0.66–1.70)	1.05 (0.65–1.70)	1.03 (0.62–1.71)
	2.5 to 5	522	35 (6.70)	1.15 (0.71–1.88)	1.15 (0.71–1.86)	0.98 (0.58–1.64)
	>5	1919	180 (9.38)	1.79 (1.20–2.65)	1.78 (1.20–2.65)	1.74 (1.14–2.64)
	*p* for trend			<0.0001	<0.0001	<0.0001
	Abdominal obesity at baseline					
	≤−2.5	181	20 (11.05)	1.00	1.00	1.00
	−2.5 to 2.5	214	57 (26.64)	2.39 (1.50–3.83)	2.46 (1.54–3.95)	2.26 (1.34–3.83)
	2.5 to 5	86	15 (17.44)	1.58 (0.85–2.93)	1.64 (0.88–3.04)	1.40 (0.70–2.81)
	>5	141	42 (29.79)	2.71 (1.67–4.39)	2.78 (1.72–4.49)	2.47 (1.43–4.28)
	*p* for trend			0.0002	0.0151	0.0051

WC: waist circumference; RR: relative risk; CI: confidence interval. * Adjusted for age at baseline. ^†^ Adjusted for variables in Model 1, as well as education level, smoking, drinking status, and physical activity, and family history of type 2 diabetes at baseline. ^‡^ Adjusted for variables in Model 2, as well as systolic blood pressure, diastolic blood pressure, levels of fasting plasma glucose, total cholesterol, triglycerides, and high-density lipoprotein cholesterol at baseline.

**Table 3:** Risk of prediabetes by abdominal obesity at baseline and follow-up.

Gender	Abdominal Obesity at Baseline	Abdominal Obesity at Follow-Up	Total	Prediabetes (*n*, %)	RR (95% CI)		
					Model 1 *	Model 2 ^†^	Model 3 ^‡^
Men	No	No	2122	257 (12.11)	1.00	1.00	1.00
	Yes	No	186	34 (18.28)	1.40 (1.01–1.94)	1.38 (1.00–1.90)	1.03 (0.69–1.53)
	No	Yes	582	103 (17.70)	1.46 (1.19–1.80)	1.46 (1.19–1.80)	1.36 (1.09–1.69)
	Yes	Yes	1016	227 (22.34)	1.73 (1.47–2.04)	1.72 (1.46–2.03)	1.35 (1.11–1.63)
Women	No	No	2857	197 (6.90)	1.00	1.00	1.00
	Yes	No	125	15 (12.00)	1.35 (0.82–2.22)	1.34 (0.81–2.20)	1.07 (0.60–1.89)
	No	Yes	566	82 (14.49)	1.90 (1.49–2.43)	1.89 (1.48–2.42)	1.60 (1.23–2.08)
	Yes	Yes	497	119 (23.94)	2.51 (1.96–3.21)	2.50 (1.95–3.19)	1.85 (1.42–2.41)

RR: relative risk; CI: confidence interval. * Adjusted for age at baseline. ^†^ Adjusted for variables in Model 1, as well as education level, smoking, drinking status, physical activity, and family history of type 2 diabetes at baseline. ^‡^ Adjusted for variables in Model 2, as well as systolic blood pressure, diastolic blood pressure, levels of fasting plasma glucose, total cholesterol, triglycerides, and high density lipoprotein cholesterol at baseline.

## Supplementary Materials

The following are available online at www.mdpi.com/1660-4601/14/10/1208/s1. Table S1: Risk of prediabetes with absolute values of WC gain.
