# Supplementary material for: Association of Waist Circumference Gain and Incident Prediabetes Defined by Fasting Glucose: A Seven-Year Longitudinal Study in Beijing, China
Source: Int J Environ Res Public Health. 2017 Oct 10;14(10):1208. doi: 10.3390/ijerph14101208 (PMC5664709; doi:10.3390/ijerph14101208)
Supplement: Supplementary file 1 [file ijerph-14-01208-s001.pdf]

Table S1. Risk of prediabetes with absolute values of WC gain.

Gender	WC Gain (cm)	Total	Prediabetes (*n*,%)	RR (95% CI)		
				Model 1 *	Model 2 †	Model 3 ‡
Men	Non-abdominal obesity at baseline					
	≤−2.5	418	48 (11.48)	1.00	1.00	1.00
	−2.5 to 2.5	673	82 (12.18)	1.12 (0.80–1.57)	1.13 (0.81–1.58)	1.18 (0.81–1.72)
	2.5 to 5	441	62 (14.06)	1.33 (0.93–1.90)	1.33 (0.93–1.89)	1.35 (0.92–1.98)
	>5	1172	168 (14.33)	1.47 (1.08–2.01)	1.50 (1.10–2.04)	1.60 (1.14–2.25)
	p for trend			0.0048	0.0031	0.0017
	Abdominal obesity at baseline					
	≤−2.5	423	80 (18.91)	1.00	1.00	1.00
	−2.5 to 2.5	365	73 (20.00)	1.05 (0.79–1.40)	1.06 (0.80–1.41)	1.13 (0.82–1.55)
	2.5 to 5	144	33 (22.92)	1.21 (0.85–1.74)	1.223 (0.86–1.75)	1.28 (0.85–1.92)
	>5	270	75 (27.78)	1.50 (1.14–1.97)	1.51 (1.15–1.99)	1.65 (1.22–2.25)
	p for trend			0.0074	0.0046	0.0018
Women	Non-abdominal obesity at baseline					
	≤−2.5	404	26 (6.44)	1.00	1.00	1.00
	−2.5 to 2.5	843	54 (6.41)	1.05 (0.67–1.64)	1.04 (0.66–1.62)	1.01 (0.63–1.62)
	2.5 to 5	558	44 (7.89)	1.39 (0.87–2.20)	1.39 (0.87–2.20)	1.28 (0.79–2.08)
	>5	1618	155 (9.58)	1.86 (1.25–2.77)	1.86 (1.25–2.77)	1.78 (1.17–2.71)
	p for trend			<0.0001	<0.0001	<0.0001
	Abdominal obesity at baseline					
	≤−2.5	211	30 (14.22)	1.00	1.00	1.00
	−2.5 to 2.5	184	47 (25.54)	1.79 (1.18–2.70)	1.81 (1.20–2.75)	1.67 (1.06–2.63)
	2.5 to 5	85	15 (17.65)	1.24 (0.71–2.19)	1.27 (0.72–2.25)	1.09 (0.58–2.04
	>5	142	42 (29.58)	2.09 (1.38–3.17)	2.13 (1.40–3.22)	1.88 (1.19–3.01)
	p for trend			0.0012	0.0008	0.0182

WC: waist circumference; RR: relative risk; CI: confidence interval. * Adjusted for age at baseline; † Adjusted for variables in model 1 as well as education level, smoking, drinking status, physical activity, family history of type 2 diabetes at baseline; ‡ Adjusted for variables in model 2 as well as systolic blood pressure, diastolic blood pressure, levels of fasting plasma glucose, total cholesterol, triglycerides and high density lipoprotein cholesterol at baseline.
